# Supplementary material for: Exploring Dolichos lablab compounds as potential inhibitors for fusion (F) protein of human metapneumovirus (HMPV): A systematic computational approach
Source: PLoS One. 2025 Sep 11;20(9):e0332170. doi: 10.1371/journal.pone.0332170 (PMC12425334; doi:10.1371/journal.pone.0332170)
Supplement: S1 Table — (DOCX) [file pone.0332170.s001.docx]

**Supplementary Table S1**. Compound Name and PubChem CID of *Dolichos lablab*.

| **Compound Name** | **PubChem CID** |
| --- | --- |
| Quercetin | 5280343 |
| Ascorbic acid | 54670067 |
| Pipecolic acid | 849 |
| D-Galacturonic Acid | 439215 |
| Cholesterol | 5997 |
| Lanosterol | 246983 |
| beta-Sitosterol | 222284 |
| Stigmasterol | 5280794 |
| (2R,3S,17R)-17-[(2S,3R,4R)-3,4-dihydroxy-6-methyl-5-methylideneheptan-2-yl]-2,3-dihydroxy-10,13-dimethyl-1,2,3,4,5,7,8,9,11,12,14,15,16,17-tetradecahydrocyclopenta[a]phenanthren-6-one | 101587911 |
| (2R,3S,5S,8S,9S,10R,13S,14S,17R)-17-[(E,2S,3R,4R)-3,4-dihydroxy-5-propan-2-ylhept-5-en-2-yl]-2,3-dihydroxy-10,13-dimethyl-1,2,3,4,5,7,8,9,11,12,14,15,16,17-tetradecahydrocyclopenta[a]phenanthren-6-one | 14607933 |
| Zeatin riboside | 6440982 |
| trans-Zeatin | 449093 |
| Palmitic acid | 985 |
| trans-Zeatin glucoside | 9842892 |
| Oleic acid | 445639 |
| Linolenic acid | 5280934 |
| Linoleic acid | 5280450 |
| (4R,5S,15R)-15-[(2S,3R,4R)-3,4-dihydroxy-6-methyl-5-methylideneheptan-2-yl]-4,5-dihydroxy-2,16-dimethyl-9-oxatetracyclo[9.7.0.02,7.012,16]octadecan-8-one | 101608671 |
| Brassinolide | 115196 |
| L-(+)-Arabinose | 439764 |
| 2'-Hydroxygenistein | 5282074 |
| Trigonelline | 5570 |
| Gibberellin A4 | 92109 |
| (1R,2R,4S,5R)-6-[(2S,3S,4R,5S,6S)-3,4,5-trihydroxy-6-(hydroxymethyl)oxan-2-yl]oxycyclohexane-1,2,3,4,5-pentol | 45109775 |
| beta-Carotene | 5280489 |
| D-Galactose | 6036 |
| 4H-Pyran-4-one, 2,3-dihydro-2,5-dihydroxy-6-methyl- | 15351062 |
| D-Glucose | 5793 |
| Psilostachyin B | 5320768 |
| Rutin | 5280805 |
| Isoquercetin | 5280804 |
| Ilicic Acid | 496073 |
| Oleanolic Acid | 10494 |
| Nandrolone | 9904 |
| Ursolic Acid | 64945 |
| Octyl decyl phthalate | 8380 |
| L-Histidine | 6274 |
| DL-Arginine | 232 |
| D-(+)-Camphor | 159055 |
| Betaine | 247 |
| N³,N⁴-Dimethyl-L-arginine | 169148 |
| N⁶-Methyladenine | 67955 |
| 9-Oxo-10(E),12(E)-octadecadienoic acid | 5283011 |
| Adenine | 190 |
| Nicotinic acid | 938 |
| 2-Hydroxyphenylalanine | 91482 |
| δ-Valerolactam | 12665 |
| 4-Piperidone | 33721 |
| L-(+)-Arginine | 6322 |
| Senkyunolide H | 13965088 |
| N-Acetyldopamine | 100526 |
| trans-3-Indoleacrylic acid | 5375048 |
| 4-Indolecarbaldehyde | 333703 |
| Ferulic acid | 445858 |
| 8-Hydroxyquinoline | 1923 |
| 4-Hydroxybenzaldehyde | 126 |
| Sinapinic acid | 637775 |
| Pyrogallol | 1057 |
| Caffeine | 2519 |
| 4-Coumaric acid | 637542 |
| Isovanillic acid | 12575 |
| Icariside B | 45783010 |
| Jasmonic acid | 5281166 |
| 7-Methyl-3-methylene-6-(3-oxobutyl)-... | 540288 |
| Maltol | 8369 |
| Butyl benzoate | 8698 |
| Scopoletin | 5280460 |
| Citral | 638011 |
| (3aR,8R,8aR,9aR)-8-Hydroxy-8a-methyl... | 23928145 |
| Ageratriol | 181557 |
| Daidzein | 5281708 |
| 9S,13R-12-Oxophytodienoic acid | 14037063 |
| Dimethomorph | 5889665 |
| 19-Norandrostenedione | 92834 |
| α-Eleostearic acid | 5282820 |
| (+/-)12(13)-DiHOME | 5282961 |
| Benzoic Acid | 15007 |
| 1-Tetradecylamine | 16217 |
| Methyl palmitate | 8181 |
| Diazinon | 3017 |
| Tributyl phosphate | 31357 |
| Nootkatone | 1268142 |
| Galaxolidone | 69131857 |
| Dibutyl phthalate | 3026 |
| Bis(2-ethylhexyl)amine | 7791 |
| Mesterolone | 15020 |
