# Supplementary material for: Exploring Dolichos lablab compounds as potential inhibitors for fusion (F) protein of human metapneumovirus (HMPV): A systematic computational approach
Source: PLoS One. 2025 Sep 11;20(9):e0332170. doi: 10.1371/journal.pone.0332170 (PMC12425334; doi:10.1371/journal.pone.0332170)
Supplement: S2 Table — (DOCX) [file pone.0332170.s002.docx]

**Supplementary Table S2**. Pharmacokinetics properties of lead compounds.

| Properties | Brassinolide | Lanosterol | Quercetin | beta-Carotene | Stigmasterol |
| --- | --- | --- | --- | --- | --- |
| **CNS Permeability (LogPS)** | -3.115 | -2.254 | -3.065 | -1.074 | -1.652 |
| **CYP2D6 substrate** | No | No | No | No | No |
| **CYP3A4 substrate** | Yes | Yes | No | Yes | Yes |
| **CYP1A2 inhibitor** | No | No | Yes | No | No |
| **CYP2C19 inhibitor** | No | No | No | No | No |
| **CYP2C9 inhibitor** | No | No | No | No | No |
| **CYP2D6 inhibitor** | No | No | No | No | No |
| **CYP3A4 inhibitor** | No | No | No | No | No |
| **Ames Toxicity** | No | No | No | No | No |
| **Hepatotoxicity** | No | No | No | No | No |
| **Acute Oral Toxicity (log(1/(mol/kg))** | 2.777 | 1.906 | 2.471 | 2.134 | 2.54 |
| **Bioavailability Score** | 0.55 | 0.55 | 0.55 | 0.55 | 0.55 |
| **Blood-Brain Barrier (BBB)** | Non-Penetrable  (High Confidence) | Penetrable  (High Confidence) | Non-Penetrable  (High Confidence) | Penetrable  (High Confidence) | Penetrable  (High Confidence) |
| **Skin Sensitisation** | No | No | No | No | No |
| **Clearance** | 4.30 | 8.64 | 8.91 | -2.04 | 7.91 |

| Properties | 2'-Hydroxygenistein | Cholesterol | Gibberellin A4 | trans-Zeatin glucoside |
| --- | --- | --- | --- | --- |
| **CNS Permeability (LogPS)** | -2.394 | -1.75 | -2.411 | -3.829 |
| **CYP2D6 substrate** | No | No | No | No |
| **CYP3A4 substrate** | Yes | Yes |  | No |
| **CYP1A2 inhibitor** | Yes | No | No | No |
| **CYP2C19 inhibitor** | No | No | No | No |
| **CYP2C9 inhibitor** | No | No | No | No |
| **CYP2D6 inhibitor** | No | No | No | No |
| **CYP3A4 inhibitor** | Yes | No | No | No |
| **Ames Toxicity** | No | No | No | No |
| **Hepatotoxicity** | No | No | Yes | No |
| **Acute Oral Toxicity (log(1/(mol/kg))** | 2.291 | 2.299 | 2.101 | 2.26 |
| **Bioavailability Score** | 0.55 | 0.55 | 0.56 | 0.17 |
| **Blood-Brain Barrier (BBB)** | Non-Penetrable  (High Confidence) | Penetrable  (High Confidence) | Penetrable  (High Confidence) | Non-Penetrable  (Low Confidence) |
| **Skin Sensitisation** | No | No | No | No |
| **Clearance** | 5.42 | 13.16 | 3.49 | 7.19 |

| Properties | Psilostachyin B | Rutin | Isoquercetin | Ilicic Acid | Oleanolic Acid |
| --- | --- | --- | --- | --- | --- |
| **CNS Permeability (LogPS)** | -2.847 | -5.178 | -4.093 | -2.133 | -1.176 |
| **CYP2D6 substrate** | No | No | No | No | No |
| **CYP3A4 substrate** | No | No | No | No | Yes |
| **CYP1A2 inhibitor** | Yes | No | No | No | No |
| **CYP2C19 inhibitor** | No | No | No | No | No |
| **CYP2C9 inhibitor** | No | No | No | No | No |
| **CYP2D6 inhibitor** | No | No | No | No | No |
| **CYP3A4 inhibitor** | No | No | No | No | No |
| **Ames Toxicity** | No | No | No | No | No |
| **Hepatotoxicity** | No | No | No | No | Yes |
| **Acute Oral Toxicity (log(1/(mol/kg))** | 2.213 | 2.491 | 2.541 | 1.807 | 2.196 |
| **Bioavailability Score** | 0.55 | 0.55 | 0.17 | 0.85 | 0.85 |
| **Blood-Brain Barrier (BBB)** | Penetrable  (High Confidence) | Non-Penetrable  (High Confidence) | Non-Penetrable  (High Confidence) | Penetrable  (High Confidence) | Penetrable  (High Confidence) |
| **Skin Sensitisation** | No | No | No | No | No |
| **Clearance** | 11.27 | 13.30 | 13.22 | 4.85 | 3.15 |

| Properties | Nandrolone | Ursolic Acid |
| --- | --- | --- |
| **CNS Permeability (LogPS)** | -2.032 | -1.187 |
| **CYP2D6 substrate** | No | No |
| **CYP3A4 substrate** | Yes | Yes |
| **CYP1A2 inhibitor** | No | No |
| **CYP2C19 inhibitor** | No | No |
| **CYP2C9 inhibitor** | No | No |
| **CYP2D6 inhibitor** | No | No |
| **CYP3A4 inhibitor** | No | No |
| **Ames Toxicity** | No | No |
| **Hepatotoxicity** | No | Yes |
| **Acute Oral Toxicity (log(1/(mol/kg))** | 2.127 | 2.346 |
| **Bioavailability Score** | 0.55 | 0.85 |
| **Blood-Brain Barrier (BBB)** | Penetrable  (High Confidence) | Penetrable  (High Confidence) |
| **Skin Sensitisation** | Yes | No |
| **Clearance** | 17.09 | 2.98 |
