# Supplementary material for: Exploring Dolichos lablab compounds as potential inhibitors for fusion (F) protein of human metapneumovirus (HMPV): A systematic computational approach
Source: PLoS One. 2025 Sep 11;20(9):e0332170. doi: 10.1371/journal.pone.0332170 (PMC12425334; doi:10.1371/journal.pone.0332170)
Supplement: Attachment — Submitted filename: Response to Reviewers.docx [file pone.0332170.s004.docx]

27 July 2025

Manuscript Number: PONE-D-25-32598

Type of manuscript: Article

Title: Exploring Dolichos lablab Compounds as Potential Inhibitors for Fusion (F) Protein of Human Metapneumovirus (HMPV): A Systematic Computational Approach.

Dear Editor,

Thank you very much for reviewing our submission titled, “**Exploring Dolichos lablab Compounds as Potential Inhibitors for Fusion (F) Protein of Human Metapneumovirus (HMPV): A Systematic Computational Approach**”. We thank all reviewers for their very constructive comments and suggestions and revised the manuscript accordingly (marked by track change function in Microsoft word in the revised manuscript). Each reviewer’s comments have been addressed below. We feel these changes have significantly strengthened this manuscript and hope that it will now be suitable for publication in biomedicines.

**Reviewer 01#**

**Q1]** First 2 paragraphs (particularly the first) of the introduction are missing key information and need reorganization. A good way to introduce is properly address respiratory infections and its burden, then viruses/ respiratory viruses. Also, we cannot address the issue of respiratory viruses without mentioning the likes of Influenza, SarsCoV2, and RSV as main causes.

**Response**: Thank you for your valuable feedback regarding the introductory section. In response, we thoroughly revised the first two paragraphs of the introduction to enhance clarity, completeness, and logical flow (**pages 2-4, lines 72-121**).

**Q2]** In the sentence “Viruses have enzymes (polymerases) that help them with genome replication….”, authors should note that not all viruses have and/or use polymerases.

**Response:** We thank the reviewer. According to the reviewer’s suggestion, we revised and removed the sentence (**page 3, lines 85, 86**).

**Q3]** In the intro, kindly use hMPV or HMPV, not both.

**Response:** Thank you for pointing this out. We revised the manuscript to ensure consistency in nomenclature by using “HMPV” uniformly throughout the introduction and the entire manuscript.

**Q4]** In the sentence “Natural products, also referred to as plant derived phytochemical…”, authors should note that natural products encompass those obtained from microbes and animals too, not only plants. Therefore, “also referred to as” can’t be used in the description.

**Response:** Thank you for the insightful observation. In response, we revised the sentence to eliminate the misleading phrasing “also referred to as” in **page 4, lines 122-124**.

**Q5]** “…phytochemicals that prevent viral reproduction and penetration…” correction: phytochemicals that prevent viral entry and replication.

**Response**: Thank you for the helpful correction. We have accordingly revised the sentence into our revised manuscript version (**page 4, lines 139, 140**).

**Q6]** It is known that HMPV F protein has the pre-Fusion and post-Fusion confirmation, with the former usually targeted for antivirals. Authors should clearly state in the intro or method that pre-fusion conformation was used.

**Response:** Thank you for the insightful comment. We revised the manuscript to clearly specify in the introduction section that the pre-fusion conformation of the HMPV fusion (F) protein was used in this study (**page 3, lines 105-108**).

**Q7]** Results section clearly stated how the 86 compounds of DL were reduced to 16 after analysis. Authors however, did not clearly mention how and why the 3 selected compounds were preferred for some of the analyses done. For example, authors commended that the 3 selected compounds have higher binding affinity than Ribavirin, but there were compounds within the list that had much better binding affinity and weren’t selected.

**Response:** We thank the reviewer for this valuable observation. While binding affinity was an important consideration in our selection process, the final three compounds, Brassinolide, Quercetin, and 2'-Hydroxygenistein, were prioritized based on a multi-criteria evaluation, which included drug-likeness (Lipinski’s rule), pharmacokinetic properties (ADME), toxicity profiles, and predicted bioactivities, in addition to molecular docking scores. Although some compounds demonstrated higher binding affinities than ribavirin, they were excluded due to suboptimal pharmacological or safety profiles (e.g., poor absorption, high toxicity, or violation of drug-likeness rules). We now revised the manuscript to clearly articulate these selection criteria in the Results section for improved clarity and justification of our compound prioritization (**page 12, lines 389-391**).

**Q8]** Authors should be consistent with keywords. Any of (a) or (b) should have the same keywords throughout the manuscript except if properly replaced first.

(a) Table 1 - Ligands; Table 2 - Compound name; Table 3 - Compounds

(b) control, Ribavirin, Ribavirin (control).

**Response**: Thank you for your valuable comment. To ensure clarity and consistency, we revised the manuscript to use uniform terminology throughout. “Compounds” is now consistently used to describe the tested molecules, and “Ribavirin” is used uniformly to refer to the reference compound in the text, tables, and figures.

**Q9]** It will be helpful if authors include Ribavirin as a control in all their analyses including in the tables shown for comparison.

**Response**: Thank you for the suggestion. In response, we have updated the relevant tables to consistently include Ribavirin, allowing for a more coherent benchmarking of the selected compounds against this reference antiviral agent.

**Q10]** “…but its clinical use is limited due to potential toxicity and lack of definitive efficacy in vivo.” Please include appropriate citation here.

**Response**: Thank you for your helpful suggestion. We included appropriate citations supporting the statement regarding the limitations of Ribavirin's clinical use due to potential toxicity and lack of definitive efficacy in vivo (**page 8, line 298**).

**Q11]** Rewrite format of references 4 and 24.

**Response**: Thank you for pointing this out. We removed reference 4 (**page 3, lines 95, 96**) and revised the formatting of reference 24 (**page 6, line 213**).

**Q12]** References 48 and 52 are not related to the statements they are cited with. Kindly review source of information.

**Response**: Thank you for pointing this out. We reviewed references 48 and 52 and revised these citations to ensure that all references accurately support the associated content (**page 13, lines 418 and 445**).

**Q13]** Figure 6C is missing the y-axis title.

Figure 6ABCD are missing the x-axis title.

Figure 7 is missing x-axis title.

**Response**: Thank you for your careful review and helpful observation. We updated Figures 6A–D and Figure 7 to include appropriate x-axis titles, and we also added the missing y-axis title to Figure 6C.

**Q14]** Authors should note that the mode of action/ target of Ribavirin is not the F protein, but the viral polymerase. Or is the author suggesting that Ribavirin potentially targets F protein of HMPV too? This doesn’t affect the analyses done with Ribavirin as a control.

**Response**: Thank you for this important observation. We acknowledge that primary mechanism of action of ribavirin involves inhibition of the viral RNA-dependent RNA polymerase, not the F protein. In our study, ribavirin was employed as a reference antiviral agent due to its reported activity against HMPV in previous studies, not to imply a direct interaction with the F protein. We clarified this point in the revised manuscript version **(page 8, lines 293-297**) to avoid any misinterpretation. This does not affect the validity of using ribavirin as a comparative control in the docking and simulation analyses.

**Q15]** Very important; Revise the quality of all the figures and ensure they meet submission standards.

**Response**: Thank you for the valuable feedback. We reviewed and revised all figures to enhance their resolution, clarity, and overall presentation quality.

**Reviewer 02#**

Summary and Strengths

This study presents a computational pipeline to identify and evaluate potential phytochemical inhibitors from Dolichos lablab targeting the HMPV fusion (F) protein. The authors integrate molecular docking, ADMET profiling, bioactivity prediction, and molecular dynamics simulations to identify promising drug candidates—Brassinolide, Quercetin, and 2'-Hydroxygenistein.

Specific strengths include:

Clear Target Rationale: The manuscript justifies the selection of the HMPV fusion protein as a therapeutic target, citing its role in viral entry and lack of existing effective inhibitors.

Use of Natural Product Library: The use of Dolichos lablab, a plant with traditional medicinal uses and known pharmacological activity, strengthens the phytochemical selection rationale.

Comprehensive Methodology:

Docking was conducted with AutoDock Vina and PyRx, with thoughtful grid definition.

ADMET and Lipinski analyses were thorough, using multiple tools (SwissADME, admetSAR, pKCSM, Deep-PK).

MD simulations were performed for 100 ns using Desmond with detailed reporting of RMSD, RMSF, Rg, SASA, and bonding interactions.

The inclusion of MMGBSA free energy calculations via PRODIGY supports the thermodynamic favorability of binding.

Comparison to a Known Control (Ribavirin): The use of Ribavirin as a benchmark adds a point of reference, improving interpretability.

Limitations and Concerns

Despite its strengths, there are several critical limitations that must be addressed before publication:

**Q1]** Over-interpretation of Computational Data

No experimental validation: All conclusions are based solely on in silico data. While the manuscript acknowledges this in the discussion, the conclusions and abstract use definitive language (e.g., “effective inhibitor,” “demonstrated promising biological activity”) that overstates the evidence.

Suggested fix: Tone down the certainty in both the abstract and conclusion, making it clear these are hypothetical inhibitors pending in vitro validation.

**Response:** Thank you for this important observation. In response, we revised the text in both conclusions and abstract sections to adopt a more cautious tone (**page 2, line 54-56 and page 15, lines 510, 512**).

**Q2]** Redundancy and Clarity

The manuscript is excessively repetitive. For instance, the results sections discussing RMSD, RMSF, and SASA repeat similar information already presented earlier. Much of this can be consolidated to improve readability.

There are also long sections describing every value in a table, which can be summarized more efficiently (e.g., in bullet format or comparative sentences).

**Response:** Thank you for your constructive feedback. In response, we consolidated overlapping information, eliminated redundancies, and restructured these sections to enhance clarity and flow (**page 13, lines 410, 411, 428, 429, 450-453**). Additionally, we summarized tabulated data more efficiently by using comparative sentences (**page 7, lines 253, 254, page 9, lines 311-320, pages 9, 10, lines 324-330, 350, page 11, lines 367-372**).

**Q3]** Misplaced Emphasis in the Introduction

The introduction spends an inordinate amount of time reviewing unrelated viruses (e.g., monkeypox, hantavirus), which dilutes focus from HMPV.

Suggested fix: Remove extraneous background on unrelated viruses and expand the part discussing existing drug discovery efforts for HMPV or other respiratory paramyxoviruses.

**Response:** Thank you for your valuable suggestion. In response, we removed extraneous background on unrelated viruses and expanded the part discussing existing drug discovery efforts for HMPV or other respiratory paramyxoviruses (**page 3, lines 76-83, 91-96**)

**Q4]** Scientific Rigor in Docking Analysis

Docking poses and interactions are described, but the manuscript does not discuss if the docking site corresponds to a functionally important site on the F protein. Do the identified binding sites overlap with known neutralizing epitopes or fusion machinery?

Suggestion: Provide structural or functional context for the binding sites—are they in the prefusion region, HR1/HR2 domains, or fusion loop?

**Response:** Thank you for your insightful suggestion. According to your suggestion, we provided structural or functional context for the binding sites (**page 8, lines 282-286**).

“The majority of the interactions were localized within regions associated with the heptad repeat domains (HR1 and HR2) and the fusion peptide, which are essential for membrane fusion and viral entry. These regions have also been implicated in the binding of neutralizing antibodies, supporting the potential functional relevance of the identified docking sites.”

**Q5]** Lack of Controls and Benchmarking

While Ribavirin is used as a control for docking energy, there is no control for the MD simulations or MMGBSA scoring other than that. Including a known non-binder or random ligand could contextualize the stability of the selected hits better.

**Response**: Thank you for this insightful comment. We acknowledge that while Ribavirin was used as a control in the docking analysis, additional controls, such as a known non-binder or a randomly selected ligand, were not included in the MD simulations or MM/GBSA scoring. We agree that incorporating such controls would provide a more rigorous comparative baseline and enhance the interpretation of the stability and binding affinity of the selected compounds. Although this was not implemented in the current study, we noted this limitation in the revised conclusion section (**page 15, lines 529-533**) and will consider it carefully in future computational and experimental validations.

**Q6]** Clarity of Figures and Tables

Figures (e.g., Fig 2, 3, 6) are referenced in the text but not included in the version I reviewed. Ensure all visual elements are clear, labeled, and aligned with journal standards.

**Response**: Thank you for pointing this out. We now ensured that all referenced figures are properly included, clearly labeled, and formatted in accordance with the journal’s submission guidelines.

Recommendation: Major Revisions

Given the extensive and well-structured computational approach, the manuscript presents interesting preliminary findings. However, I recommend major revisions before this work can be considered for publication. Specifically:

**Q1]** Revise the abstract and conclusion to avoid overstatements.

**Response:** Thank you for this important suggestion. We carefully revised both the abstract and conclusion to avoid overstatements and to more accurately reflect the computational nature of our study.

**Q2]** Reduce redundancy and improve clarity in the writing.

**Response:** Thank you for your observation. We thoroughly revised the manuscript to reduce redundancy and improve clarity throughout the text. Repetitive statements have been removed or consolidated, and several sections have been rewritten to enhance readability and maintain a clear and concise presentation of the findings.

**Q3]** Improve discussion of the docking site's biological relevance.

**Response:** Thank you for this valuable comment. We have expanded the discussion to provide greater insight into the biological relevance of the docking site (**page 8, lines 282-286**).

**Q4]** Remove extraneous introductory background.

**Response:** Thank you for the helpful suggestion. We revised the introduction to remove extraneous background information that is not directly relevant to the study’s focus (**page 3, lines 76-83, 91-96**)

**Q5]** Ensure all compound IDs, docking poses, and data are accurately labeled and interpreted.

**Response**: Thank you for this important observation. We carefully reviewed all compound identifiers, docking poses, and associated data to ensure they are accurately labeled, consistently presented, and correctly interpreted throughout the manuscript.

**Q6]** Consider additional computational validation (e.g., benchmarking with known ligands or adding entropy calculations).

**Response:** Thank you for this valuable recommendation. We added additional computational validation like benchmarking with known ligands into our revised manuscript version (**page 8, lines 259-271**).

**Reviewer 03#**

Strengths:

Scope and Relevance:

The study investigates the absence of targeted therapies for HMPV by conducting a screening of phytochemicals derived from Dolichos lablab. This is both novel and pertinent to the global respiratory health. It also incorporates ligand mining, molecular docking, ADMET profiling, bioactivity prediction, molecular dynamics (100 ns), and MMGBSA calculations, offering a comprehensive computational analysis.

The three compounds Brassinolide, Quercetin, and 2-Hydroxygenistein were prominently identified as leading candidates based on consistent outcomes from docking, ADMET, and MD simulations. Comprehensive tables and figures enhance transparency and replicability. The incorporation of MMGBSA values and protein-ligand interaction maps significantly augments scientific rigour.

Methodology:

The author utilized AutoDock Vina, SwissADME, admetSAR, Molinspiration, and Desmond effectively. They also made use of 100 ns molecular dynamics simulations, which are standard or above average in duration for preliminary in silico investigations.

The MMGBSA computation utilizing PRODIGY facilitates energetic validation of the docking results.

Presentation:

The methodologies and software are documented with their respective versions and settings.

Also, the data, tables and figures are appropriately labelled.

Suggestions:

**Q1]** Incorporate RMSD-based validation or re-docking of established inhibitors to ascertain docking precision.

**Response:** Thank you for this valuable suggestion. According to your suggestion, we incorporated RMSD-based re-docking validation of established inhibitors to ascertain docking precision (**page 8, lines 259-271**)

**Q2]** Please describe the filtering process.

**Response:** Thank you for your comment. In this study, molecular docking was performed initially on all candidate compounds to evaluate their binding affinity to the HMPV F protein. Following docking, a filtering process was applied to prioritize compounds based on multiple criteria, including binding energy, pharmacokinetic properties (such as ADMET profiles), drug-likeness (using Lipinski’s Rule of Five and related filters), and toxicity predictions. Compounds showing favorable docking scores but poor drug-like or safety profiles were excluded from downstream analyses. This sequential approach allowed us to identify a focused set of lead compounds with both strong binding potential and acceptable pharmacological characteristics.

**Q3]** Can you explain the necessity for subsequent experimental investigations in vitro and in vivo?

**Response:** Thank you for the question. While our study provides valuable insights through computational methods, such as molecular docking, pharmacokinetic profiling, and molecular dynamics simulations, these predictions remain hypothetical until experimentally validated. In vitro studies are essential to confirm the actual binding affinity, antiviral activity, and cytotoxicity of the selected compounds under controlled laboratory conditions. In vivo studies are equally important to assess pharmacodynamics, bioavailability, metabolism, and potential toxicity within a biological system. Together, these experimental investigations are critical to translate in silico predictions into clinically relevant therapeutic candidates and to determine their real-world efficacy and safety.

**Q4]** Also you should examine Lipinski's rule infractions and their impact on translational viability.

**Response:** Thank you for the valuable comment. We have examined the selected compounds for any infractions of Lipinski’s Rule of Five and assessed how these violations might influence their pharmacokinetic properties and overall translational viability (**pages 9, 10, 11, lines 310-356, 365-381**).
