# Supplementary material for: Exploring Dolichos lablab compounds as potential inhibitors for fusion (F) protein of human metapneumovirus (HMPV): A systematic computational approach
Source: PLoS One. 2025 Sep 11;20(9):e0332170. doi: 10.1371/journal.pone.0332170 (PMC12425334; doi:10.1371/journal.pone.0332170)
Supplement: Attachment — Submitted filename: Response_to_Reviewers_auresp_2.docx [file pone.0332170.s005.docx]

21 August 2025

Manuscript Number: PONE-D-25-32598R1

Type of manuscript: Research Article

Title: Exploring Dolichos lablab Compounds as Potential Inhibitors for Fusion (F) Protein of Human Metapneumovirus (HMPV): A Systematic Computational Approach.

Dear Editor,

Thank you very much for reviewing our submission titled, “**Exploring Dolichos lablab Compounds as Potential Inhibitors for Fusion (F) Protein of Human Metapneumovirus (HMPV): A Systematic Computational Approach**”. We thank all reviewers for their very constructive comments and suggestions and revised the manuscript accordingly (marked by track change function in Microsoft word in the revised manuscript). Each reviewer’s comments have been addressed below. We feel these changes have significantly strengthened this manuscript and hope that it will now be suitable for publication in biomedicines.

**Editor Comments**

**Q1]** Please review your reference list to ensure that it is complete and correct. If you have cited papers that have been retracted, please include the rationale for doing so in the manuscript text, or remove these references and replace them with relevant current references. Any changes to the reference list should be mentioned in the rebuttal letter that accompanies your revised manuscript. If you need to cite a retracted article, indicate the article’s retracted status in the References list and also include a citation and full reference for the retraction notice.

**Response**: Thank you for your guidance regarding the reference list. We thoroughly reviewed all references to confirm their accuracy, completeness, and relevance. No retracted articles were identified, and therefore no changes to the reference list were required. The references remain scientifically current and appropriate to the manuscript content.

**Additional Editor Comments:**

**Q1]** In lines 361-382, the authors have subtitled this section including the Table 4 as "Bioactivity". However, the only parameters presented there are TPSA, Molar volume and Rotatable bonds, which are understandably, physicochemical parameters for predicting polarity, size and flexibility of the molecules respectively. The rationale for tagging these "Bioactivity" remains confusing.

**Response:** Thank you for this observation. We agree that the parameters presented in Table 4—TPSA, molar volume, and rotatable bonds—are physicochemical descriptors primarily used to predict molecular polarity, size, and conformational flexibility, rather than direct measures of biological activity. The section was originally titled “Bioactivity” to indicate that these descriptors are often evaluated in early-stage drug-likeness and bioavailability screening, as they influence membrane permeability and receptor binding potential.

However, we acknowledge that the term “Bioactivity” could be misleading in this context. To improve clarity and scientific accuracy, we changed the section title from “Bioactivity” to “**Physicochemical Properties Related to Drug-Likeness**” in the revised manuscript (**page 6, lines 212, 215 and 216, pages 11, 12, lines 362, 364, 366, 387, 390, and 395**).

**Q2]** Moreover, the data are mere predictions which require extensive experimental validation for certainty. I strongly recommend that these sections be reconstructed and the superfluous statements be modified. The title should also indicate prediction since no experimental results have been provided.

**Response:** Thank you for your insightful suggestions. According to your suggestions, we reconstructed and modified superfluous statements into our revised manuscript (**page 12, lines 394, 400-402**) as well as we indicated prediction into title section (**page 11, line 362**).
